# Randomly and Non-Randomly Missing Renal Function Data in the Strong Heart Study: A Comparison of Imputation Methods

**DOI:** 10.1371/journal.pone.0138923

**Published:** 2015-09-28

**Authors:** Nawar Shara, Sayf A. Yassin, Eduardas Valaitis, Hong Wang, Barbara V. Howard, Wenyu Wang, Elisa T. Lee, Jason G. Umans

**Affiliations:** 1 MedStar Health Research Institute, Hyattsville, Maryland, United States of America; 2 Georgetown-Howard Universities Center for Clinical and Translational Science, Washington, District of Columbia, United States of America; 3 Department of Mathematics and Statistics, American University, Washington, District of Columbia, United States of America; 4 College of Public Health, University of Oklahoma, Oklahoma City, Oklahoma, United States of America; Nankai University, CHINA

## Abstract

Kidney and cardiovascular disease are widespread among populations with high prevalence of diabetes, such as American Indians participating in the Strong Heart Study (SHS). Studying these conditions simultaneously in longitudinal studies is challenging, because the morbidity and mortality associated with these diseases result in missing data, and these data are likely not missing at random. When such data are merely excluded, study findings may be compromised. In this article, a subset of 2264 participants with complete renal function data from Strong Heart Exams 1 (1989–1991), 2 (1993–1995), and 3 (1998–1999) was used to examine the performance of five methods used to impute missing data: listwise deletion, mean of serial measures, adjacent value, multiple imputation, and pattern-mixture. Three missing at random models and one non-missing at random model were used to compare the performance of the imputation techniques on randomly and non-randomly missing data. The pattern-mixture method was found to perform best for imputing renal function data that were not missing at random. Determining whether data are missing at random or not can help in choosing the imputation method that will provide the most accurate results.

## Introduction

Missing medical data are common in epidemiologic studies. This problem is exacerbated in longitudinal studies, where missing data increase over time, sometimes compromising results [1]. Because it can be difficult or impossible to verify whether data are missing at random (MAR) or are related to the outcome of interest [2], some studies ignore missing data by dropping missing observations from the data set. Although such listwise deletion (LD) is sometimes the simplest or only way to conduct an analysis, this method can lead to inaccurate conclusions. In such cases, investigators impute missing values to generate complete data for a key variable or to maximize power for an intent-to-treat study. The objective of this article is to aid researchers in selecting the imputation method that will provide the most valid estimates by simulating the nature of missing data in the Strong Heart Study (SHS) and testing differing remedial measures. In this article, the importance of understanding the reason missing values may arise in the data is highlighted, and the measures most appropriate for each context are discussed.

Missing data can be classified in one of three ways: missing completely at random (MCAR), missing at random (MAR), and not missing at random (NMAR). When the probability that a value is missing is statistically independent of its own hidden value and the value of all other variables, then the data are considered MCAR. Because of this independence, the bias resulting from the missing data is mitigated, but because most software will automatically apply listwise deletion (LD) to observations with missing data, reduction in power and information loss can be substantial. When the probability that a value is missing is correlated with the values of other variables, but is independent of its own hidden value, then the data are considered MAR. Finally, if the probability that a value is missing is correlated with its own value, then the data are considered NMAR. Understanding why data are missing in a data set is an important factor in choosing the remedial measure to be used for imputing the missing data. While general rules regarding imputation method applications apply, the appropriateness of any method is largely determined in each specific case based on numerous factors, including the nature of the missing data. In this article, we will investigate this issue using data from the SHS, a longitudinal study of American Indians.

Kidney disease is widespread among populations with high prevalence of diabetes. Individuals with chronic kidney disease (CKD) are at elevated risk for all-cause and cardiovascular disease (CVD) mortality [3–9], and an adverse CVD risk factor profile is associated with declining kidney function [10]. Occurrence of diminishing renal function along with worsening CVD risk factors is a phenomenon challenging to longitudinal study of these diseases in populations, such as American Indians, in which both conditions are common. The morbidity and mortality associated with these diseases result in large amounts of missing data, and these data are likely NMAR.

The SHS population has high rates of CVD, diabetes, and renal disease. Data on serum creatinine (Scr), a screening test for kidney function, are missing at varying rates across the three phases of the SHS study, in some cases probably for non-random reasons. Therefore, after selecting a subset of patients with full longitudinal data, we simulated missing data using four different methods. We then applied five different remedial measures to deal with the missing values. A Cox survival regression was executed on the full data set, predicting time to a hard atherosclerotic cardiovascular disease event based on Scr. A Cox regression model was then performed on the imputed data sets, and the hazard ratios for Scr values at each exam were compared with the hazard ratios of the first. We hypothesized that the pattern-mixture (PM) method would generate hazard ratios closest to those in the complete set.

## Methods

### Study population

The SHS was initiated in 1988 to investigate CVD and its risk factors in American Indians from 13 tribes in Arizona, Oklahoma, and North and South Dakota. The SHS design and methods have been published [11–12]. The SHS was approved by the Oklahoma Center Indian Health Service institutional review board (IRB), the Dakota Center Indian Health Service IRB, the Arizona Center Indian Health Service IRB, and the MedStar Health Research Institute IRB. In addition, this study was approved by the American Indian communities. All data were anonymized and de-identified before the analyses. This cohort of 4549 American Indians includes men and women ages 45–74 years seen at the first (1989–1991), second (1993–1995), and third (1998–1999) exams. Participants receiving dialysis or who had a kidney transplant were eliminated from the data set. Of the 4549 SHS participants at baseline, 3605 were alive at Exam 3, and 2219 (62%) were women. A subset of 2264 participants with complete renal function data at all three exams was used for the current analyses.

### Renal function measures

Scr was assayed by a single core laboratory using automated alkaline picrate rate methodology [12]. Urinary creatinine was measured at all three exams [10,13].

### Cardiovascular and diabetes surveillance

CVD surveillance for nonfatal and fatal clinical events occurred throughout the follow up and is complete through December 31, 2003 [14]. Criteria used to define definite fatal myocardial infarction, stroke, coronary heart disease, and nonfatal CVD have been published [15], as have methods for ascertaining incident CVD events [13, 16–17]. Incident diabetes was identified by self-report, use of hypoglycemic agents, or fasting glucose ≥126 mg/dl [18].

### Creation of missing data models

The complete SHS data set included age, gender, history of diabetes, CVD status, and three serial measures of Scr. The outcome variable of interest was CVD. Four models with randomly (Models 1–3) and non-randomly (Model 4) missing data were created from the complete data set using the algorithms described below.

#### Model 1, Base data with MAR data

In Model 1, the data were missing at random. This model was created from the complete data set.

#### Model 2, Autoregressive MAR data

Let *M* be the matrix that represents the missing data, so a value of 0 indicates that the observation exists and 1 indicates that it is missing. Then consider the *Y*_*p*_ matrix, which consists of Scr measurements on the 3605 subjects:

$$Y_{p}=\left[\begin{array}{ccc} Scr_{11} & Scr_{12} & Scr_{13}\\ \vdots & & \\ Scr_{n1} & Scr_{n2} & Scr_{nk} \end{array}\right]$$

Let *M*_*p*_ be the matrix of missing data associated with *Y*_*p*._ We proposed the following autoregressive MAR generating mechanism: *P*(*M*_*p*_[*i*,*j*] = 1) = *f*(*M*_*p*_[*i*,*j* − 1]), (*Model 1*); where *M*_*p*_[*i*,*j*] corresponds to the entry in the row *I* and column *j* in *M*. The probability that a subject has a missing value at stage *j* depends on whether the subject had a missing value in stage *j –*1. Thus, missing Scr data at Exam 3 are not independent of missing Scr data at Exam 2 and any algorithm generating MAR data should account for that, even though missing Scr data at Exam 2 are independent of missing data at Exam 1. When fitting an autoregressive model of the form *P*(*M*_*p*_[*i*,2] = 1) = *α* + *β* × 1{*M*_*p*_[*i*,1] = 1}, the slope coefficient has a t-value of 1.48 and thus is not significant at the 10% alpha level. Missing values at Exam 1 were selected at random with a Bernouli random generator.

#### Model 3, Autoregressive MAR data augmented with gender and age

**For gender,** let X be a binary vector denoting gender, with women having an entry of 0 and men an entry of 1. Women and men appear to have different rates of missing data. At Exams 2 and 3, women have significantly lower rates of missing data than men (all *P* values <0.01). At Exam 1, the significant difference between genders was not observed (p>0.05).

Thus, we proposed the following algorithm to generate values for Model 3 *P*(*M*_*p*_[*i*,*j*] = 1) = *α* + *β*_*g*_ *X* + *β*_*m*_1{*M*_*p*_[*i*,*j* − 1] = 1}, where 1{*M*_*p*_[*i*,*j* − 1] = 1} is an indicator function with a value of 1 when Scr is missing in the previous exam. We did not apply this model to Exam 1 missing data, because no significant difference between genders was observed. The following generalized linear models with a binary response variable for the rate of missing data and an identity link were fitted using the Broyden-Fletcher-Goldfarb-Shanno (BFGS) method [19]. The BFGS method solves an unconstrained nonlinear optimization problem using gradient descent. It is a member of a broad class of hill-climbing optimization techniques. We applied it to predict the rate of missing data at Exams 2 and 3 as follows:

**Exam 2**: *P*(*M*_*p*_[*i*,2] = 1) = .089+.036*X*; gender coefficient t-value is 4.2;

**Exam 3:** *P*(*M*_*p*_[*i*,3] = 1) = .082+.029*X* + .353×1{*M*_*p*_[*i*,2] = 1}; gender coefficient has a t statistic of 3.44, while the autoregressive coefficient has a t-value of 21.75.

**Age** is a variable that influences the rate of missing observations. The elderly may experience difficulty getting to the testing site, may move to retirement homes or hospices, or may die, thus missing exams. The relationship between the probability of missing data and participant age at Exams 1 and 2 was weak and unexpectedly U-shaped, while the probability of a relationship between participant age and missing data at Exam 3 was a more pronounced U-shape. One explanation is that younger participants may drop out more frequently because of employment or relative lack of concern for personal health. The following formulas define Model 3, using age (denoted by *Y*) and previous missing data as explanatory variables (all of the coefficients were significant, thus we excluded the t-values).

**Exam 2:** $P\left(M_{p}\left[i,2\right]=1\right)=1.93+.030\times Y-.461\times \sqrt{Y}$,

**Exam3:** *P*(*M*_*p*_[*i*,3] = 1) = 3.62 + .065×*Y* − .953×ln(*Y*) + .355×1{*M*_*p*_[*i*,2] = 1}.

Finally, including all the explanatory factors, we propose Model 3 with **gender and age** as a MAR algorithm:

**Exam 2:** $P\left(M_{p}\left[i,2\right]=1\right)=1.80+.035\times X+.028\times Y-.44\times \sqrt{Y}$,

**Exam 3:** *P*(*M*_*p*_[*i*,3] = 1) = 3.22 + .030×*X* + .059×*Y* − .863×ln(*Y*) + .353×1{*M*_*p*_[*i*,2] = 1}. Adding gender does not dramatically alter the age and the previous missing data coefficients from this model, yet the likelihood-ratio tests show that adding age decreases residual deviance in Exams 2 and 3 significantly. In this analysis, Model 3 with gender and age was the one used to test the performance of the different imputation methods on autoregressive missing data augmented with covariates.

#### Model 4. Empirical NMAR data

Similar to the empirical models for MAR data (Models 1–3), the following model estimates two potential NMAR mechanisms for our complete Scr data set. The algorithm used was developed by Troxel et al. [20]. This algorithm assumes that data are multivariate normal and *f(Y*_*t*_ *|Y*_*t-1*_,*Y*_*t-2*_,*…) = f(Y*_*t*_ *|Y*_*t-1*_ *)*.

Let π_*i*_ denote the probability that the observation is missing at stage *i*. We then fit the following model for women and men separately: logit(π_*i*_) = *α*+***β*** *y*_*ij*_ ; where *y*_*ij*_ was the Scr value in phase *i* for subject *j*. We estimated the parameters above using the Likelihood function described by Troxel et al. Using the S-Plus library MASS and the function OPTIM [21] to maximize the likelihood, we obtained standard errors of the estimates by retaining the Hessian matrix, inverting it, and taking the square root of the diagonal.

#### Empirical NMAR data model for women and men

The algorithm used in this model was developed by Troxel et al. [20], which assumes that the data are multivariate normal and *f(Y*_*t*_ *|Y*_*t-1*_,*Y*_*t-2*_,*…) = f(Y*_*t*_ *|Y*_*t-1*_ *)*.

The Troxel method: Let π_*i*_ denote the probability that the observation is missing at stage *i*. We will then fit the following model for women and men separately: logit(π_*i*_) = *α*+***β*** *y*_*ij*_ ; where *y*_*ij*_ is the Scr value in phase *i* for subject *j*. We will estimate the parameters above by using the Likelihood function [20]. The parameter estimates and their standard errors (in parentheses) were obtained for women and men for the equation above.

For women, the parameter estimates were significant, but for men, the intercept was significant. At Exam 2, the ***β*** estimate was not significant at the 5% significance level, and at Exam 3 it was marginally significant at that level. The probability of missing data is, therefore, less dependent on Scr values for men than for women.

The fitted missing data probability functions were used to generate the last missing value mechanism. Although the fitted NMAR model was not significant for men at Exam 2, it is important to examine the effects of missing data on the outcome measures.

The four models are summarized below:

Model 1: Base data with MAR data

Model 2: Autoregressive MAR data

Model 3: Autoregressive MAR data +gender + age

Model 4. Empirical NMAR data using the Troxel et al. algorithm.

Missing data were then replaced in each model using each of five imputation methods (LD, mean of serial measures, adjacent value [AV], multiple imputation [MI], and PM) to compare the efficacy of these methods in providing Scr values for randomly and non-randomly missing data.

### Imputation methods

The five imputation methods used to fill in missing Scr values are described:

■ *Listwise deletion* (LD), where observations with missing data are dropped. This method should only be used with MAR data, as it can generate biased data. This method reduces the sample size.
■ *Imputation using the mean* (mean of serial measures), where the overall variable mean is used to impute cross-sectional data. Use of this method is limited to continuous data. In this study, this method was used with mean Scr values from the corresponding exam to impute missing Scr values.
■ *AV*, where missing data are replaced by using the most adjacent value. In this study, these came from either the previous or subsequent exam. This method is used to impute missing longitudinal data. In this study, it was assumed that no change in kidney function occurred after a clinical or sub-clinical event. This method may bias results because of early dropouts who have less favorable measures [22].
■ *MI*, where multiple single imputations are carried out simultaneously on the incomplete data set to obtain a fitted model with differing parameter estimates. Missing data are assumed to be MAR.
■ *PM [23–25]*, where assumptions are applied to the missing data using MI techniques. This method specifies thresholds to restrict the imputed values. The assumption in this study was that patients with lower Scr values were more likely to have missing values. Therefore, the restricting upper-bound thresholds selected were the 10^th^, 25^th^, and 50^th^ percentiles of the predicted Scr values.

### Statistical Analysis

Baseline characteristics were provided for the SHS participants with complete renal data. Means with corresponding 95% confidence intervals (CIs) of Scr were generated by each imputation method and compared with the complete data set for each of the generated missing data models. Cox proportional hazard models were used to examine associations between CVD risk and imputed Scr in the four models. Hazard ratios (HRs) and 95% CIs were calculated and adjusted for age, gender, and diabetes status.

The five imputation methods were compared to determine a) differences in distributions between the imputed data sets and the complete data set as measured by the mean and 95% CIs, b) discrepancies in estimates of the adjusted HRs of incident CVD and limits of the 95% CIs for each imputed data set versus the complete set at each exam using a non-time-dependent covariate Cox proportional model, and c) the significance of the models examined in each imputed data set compared with the complete data set using a time-dependent covariate Cox proportional model. The method that provided HR estimates closest to those generated by the complete data set was considered to perform the best.

## Results

Among the entire SHS cohort of 4549 subjects, the percentage of missing data at Exams 1, 2, and 3 was 3.8%, 22.3%, and 32.5%, respectively. The generated rates of missing Scr data for the 2264 participants in Models 1, 2, 3, and 4 were approximately 20%, 30%, and 40% at Exams 1, 2, and 3, respectively.

In the 2264 SHS participants with complete Scr data for all three exams, mean baseline Scr was 0.88 mg/ml (standard deviation [SD] = 0.3 mg/ml), and mean age was 54.9 years (SD = 7.4 years). Sixty-four percent of participants were female, 37.3% had diabetes, and 4.6% had prevalent CVD. During a median 10 years of follow up (Exam 1 to December 31, 2003), 447 (19.7%) experienced a CVD event (Table 1).

**Table 1:** Baseline Characteristics of Strong Heart Study Participants with Complete Scr Data at All Three Exams (*N* = 2,264).

Variable	N	Mean (SD)
Scr	2,264	0.88±0.25
Age	2,264	54.9±7.4
Female	1451	64.1%
Diabetes	845	37.3%
Prevalent CVD	104	4.6%
Incident CVD by 2003	447	19.7%

Abbreviations: CVD = cardiovascular disease; Scr = serum creatinine.

The distribution of Scr in the four models is presented for each of the five imputation methods (Table 2). Models 1, 2, and 3 represent MAR data, while Model 4 represents the NMAR data. All the imputation methods underestimated the mean, especially for Model 4 at Exam 3, in which the rate of missing data was approximately 40%. LD performed slightly better than the other imputation methods in Models 1, 2, and 3, but underestimated the mean and SD in Model 4 at Exams 2 and 3. Imputation using the mean also performed well in Models 1, 2, and 3 but, like the LD method, underestimated the mean and SD in Model 4 at Exams 2 and 3. Imputation using the AV did not perform as well as LD in Models 1, 2, and 3, but it was slightly better in not underestimating the mean and SD in Model 4 at Exams 2 and 3 than the LD and MI methods were. Imputation using MI overestimated the mean in Models 1, 2, and 3 at Exam 3 and underestimated the mean and SD in Model 4 at Exam 3. The PM method at the 10^th^, 25^th^, and 50^th^ percentiles of the Scr data estimated the mean in Model 4 at Exams 2 and 3 across all levels of percentiles better than the other imputation methods did. Additionally, the higher percentiles seemed to provide estimates closer to those made with the complete data set.

**Table 2:** Mean and SD of Scr Values Stratified by Imputation Method and Model.

Exam 1	Complete Data Scr mean (sd): **0.88 (0.25)**			
	Missing Data Generation Method			
Imputation method	Data with Randomly Missing Values (Model 1)	Autoregressive Missing (Model 2)	Autoregressive w/ Gender and Age (Model 3)	Troxel Algorithm (NMAR Data; Model 4)
LD	0.87 (0.26)	0.87 (0.18)	0.88 (0.27)	0.88 (0.27)
Mean	0.87 (0.24)	0.87 (0.16)	0.88 (0.24)	0.88 (0.24)
AV	0.87 (0.26)	0.87 (0.18)	0.88 (0.27)	0.88 (0.27)
MI	0.87 (0.25)	0.87 (0.17)	0.87 (0.25)	0.88 (0.25)
PM (10^th^ percentile)	0.88 (0.26)	0.87 (0.17)	0.89 (0.25)	0.88 (0.25)
PM (25^th^ percentile)	0.88 (0.25)	0.88 (0.17)	0.89 (0.25)	0.89 (0.26)
PM (50^th^ percentile)	0.88 (0.26)	0.88 (0.17)	0.89 (0.25)	0.89 (0.26)
**Exam 2**	Complete Data Scr mean (sd): **0.90 (0.44)**			
	Missing Data Generation Method			
Imputation method	Data with Randomly Missing Values (Model 1)	Autoregressive Missing (Model 2)	Autoregressive w/ Gender and Age (Model 3)	Troxel Algorithm (NMAR Data; Model 4)
LD	0.90 (0.47)	0.89 (0.35)	0.90 (0.45)	0.83 (0.19)
Mean	0.90 (0.39)	0.89 (0.29)	0.90 (0.38)	0.83 (0.16)
AV	0.89 (0.43)	0.89 (0.32)	0.89 (0.41)	0.86 (0.28)
MI	0.91 (0.45)	0.89 (0.31)	0.91 (0.39)	0.84 (0.19)
PM (10^th^ percentile)	0.95 (0.45)	0.93 (0.32)	0.95 (0.41)	0.86 (0.19)
PM (25^th^ percentile)	0.97 (0.46)	0.95 (0.32)	0.97 (0.41)	0.88 (0.19)
PM (50^th^ percentile)	0.98 (0.46)	0.96 (0.33)	0.99 (0.42)	0.90 (0.21)
**Exam 3**	Complete Data Scr mean (sd): **0.94 (0.88)**			
	Missing Data Generation Method			
Imputation method	Data with Randomly Missing Values (Model 1)	Autoregressive Missing (Model 2)	Autoregressive w/ Gender and Age (Model 3)	Troxel Algorithm (NMAR Data; Model 4)
LD	0.95 (0.94)	0.94 (0.85)	0.93 (0.85)	0.76 (0.17)
Mean	0.95 (0.73)	0.94 (0.67)	0.93 (0.67)	0.76 (0.13)
AV	0.93 (0.81)	0.93 (0.72)	0.92 (0.74)	0.82 (0.26)
MI	1.03 (0.86)	1.00 (0.76)	1.00 (0.77)	0.79 (0.17)
PM (10^th^ percentile)	1.14 (0.89)	1.09 (0.79)	1.12 (0.83)	0.81 (0.17)
PM (25^th^ percentile)	1.16 (0.89)	1.12 (0.80)	1.13 (0.82)	0.83 (0.17)
PM (50^th^ percentile)	1.19 (0.91)	1.13 (0.82)	1.16 (0.84)	0.85 (0.19)

Abbreviations: AV = imputation using adjacent value; LD = listwise deletion; Mean = imputation using the mean; MI = multiple imputation; NMAR = not missing at random; PM = pattern mixture.

Model 1 = data with randomly missing values

Model 2 = autoregressive missing

Model 3 = autoregressive +gender + age

Model 4 = NMAR data.

The imputation techniques also were compared with respect to adjusted HRs and 95% CIs for CVD risk at Exams 1, 2, and 3 across the four models (Table 3). The complete case data showed significant relations between Scr and CVD risk only at Exam 2. Performance of the imputation methods varied with the different data sets. At Exam 1, all the imputation methods gave similar estimates of HRs. All the imputation methods showed significant relations between imputed Scr and CVD risk in Model 2 at Exam I. At Exam 2, all the imputation methods yielded significant results between the imputed Scr values and CVD risk in Models 2 and 3. The biggest difference in the results was found in Model 4 at Exam 2. A non-significant protective effect (i.e., HRs <1) was found in Model 4 using the LD and mean imputation methods. At Exam 3, all of the imputation methods yielded similar results in Models 1, 2, and 3, but overestimated the hazard ratio in Model 4, compared with the complete data set. However, the estimated HRs using the PM and AV methods were closer to the HRs from the complete data set.

**Table 3:** Adjusted Hazard Ratios With 95% Confidence Intervals for Cardiovascular Disease Risk.

Exam 1	Complete Data Scr HR (95% CI): **1.15 (0.87–1.53)**			
	Missing Data Generation Method			
Imputation method	Data with Randomly Missing Values(Model 1)	Autoregressive Missing (Model 2)	Autoregressive w/ Gender and Age (Model 3)	Troxel Algorithm (NMAR Data; Model 4)
LD	1.05 (0.73–1.51)	2.21 (1.31–3.67)	1.16 (0.86–1.55)	1.17 (0.88–1.55)
Mean	1.06 (0.75–1.51)	2.19 (1.31–3.67)	1.13 (0.83–1.54)	1.16 (0.87–1.54)
AV	1.05 (0.73–1.51)	2.21 (1.30–3.76)	1.16 (0.86–1.55)	1.17 (0.88–1.55)
MI	1.11 (0.82–1.51)	1.98 (1.18–3.33)	1.16 (0.87–1.54)	1.15 (0.87–1.54)
PM (10^th^ percentile)	1.08 (0.78–1.49)	2.05 (1.23–3.41)	1.13 (0.84–1.53)	1.16 (0.86–1.56)
PM (25^th^ percentile)	1.09 (0.80–1.49)	2.11 (1.26–3.52)	1.15 (0.85–1.56)	1.14 (0.85–1.54)
PM (50^th^ percentile)	1.10 (0.80–1.51)	2.21 (1.30–3.75)	1.17 (0.88–1.55)	1.19 (0.91–1.55)
**Exam 2**	Complete Data Scr HR (95% CI): **1.17 (1.01–1.35)**			
	Missing Data Generation Method			
Imputation method	Data with Randomly Missing Values (Model 1)	Autoregressive Missing (Model 2)	Autoregressive w/ Gender and Age (Model 3)	Troxel Algorithm(NMAR Data; Model 4)
LD	1.14 (0.96–1.35)	1.37 (1.11–1.68)	1.23 (1.08–1.41)	0.66 (0.32–1.47)
Mean	1.14 (0.97–1.35)	1.34 (1.09–1.65)	1.23 (1.08–1.40)	0.69 (0.32–1.35)
AV	1.12 (0.94–1.33)	1.40 (1.16–1.69)	1.23 (1.09–1.40)	1.09 (0.76–1.55)
MI	1.10 (0.92–1.31)	1.34 (1.08–1.66)	1.22 (1.06–1.40)	1.01 (0.53–1.95)
PM (10^th^ percentile)	1.07 (0.9–1.30)	1.38 (1.14–1.67)	1.24 (1.09–1.40)	1.19 (0.59–2.42)
PM (25^th^ percentile)	1.07 (0.88–1.29)	1.40 (1.16–1.68)	1.23 (1.08–1.40)	1.05 (0.55–1.98)
PM (50^th^ percentile)	1.09 (0.91–1.31)	1.40 (1.15–1.70)	1.23 (1.08–1.40)	1.16 (0.66–2.02)
**Exam 3**	Complete Data Scr HR (95% CI): 1.**10 (0.97–1.25)**			
	Missing Data Generation Method			
Imputation method	Data with Randomly Missing Values (Model 1)	Autoregressive Missing (Model 2)	Autoregressive w/ Gender and Age (Model 3)	Troxel Algorithm (NMAR Data; Model 4)
LD	1.09 (0.94–1.26)	1.12 (0.95–1.31)	1.07 (0.89–1.29)	1.79 (0.51–6.23)
Mean	1.10 (0.94–1.27)	1.10 (0.94–1.28)	1.07 (0.90–1.28)	1.76 (0.54–5.78)
AV	1.08 (0.93–1.25)	1.13 (0.98–1.30)	1.12 (0.96–1.30)	1.39 (0.93–2.06)
MI	1.04 (0.89–1.21)	1.13 (0.99–1.30)	1.11 (0.97–1.27)	1.87 (0.70–4.97)
PM (10^th^ percentile)	1.00 (0.8–1.20)	1.11 (0.95–1.31)	1.11 (0.96–1.28)	1.52 (0.62–3.76)
PM (25^th^ percentile)	1.02 (0.86–1.20)	1.13 (0.98–1.31)	1.12 (0.98–1.27)	1.38 (0.54–3.54)
PM (50^th^ percentile)	1.01 (0.86–1.19)	1.13 (0.98–1.30)	1.11 (0.98–1.27)	1.37 (0.58–3.26)

^&^ Cox proportional regression models adjusted for age, gender, and diabetes.

*Significant at 5%.

Abbreviations: LD = listwise deletion; Mean = imputation using the mean; AV = imputation using adjacent value; MI = multiple imputation; NMAR = not missing at random; PM = pattern mixture.

Complete Data: data with no missing values

Model 1: data with randomly missing values

Model 2: autoregressive missing

Model 3: autoregressive +gender + age

Model 4: NMAR data.

In a time-dependent covariate Cox model (Table 4), in which we did not break down the data into the three exam periods, the adjusted HRs with 95% CIs for CVD risk were stronger for all the imputation methods in Model 4, compared with the complete data set. In Model 4, all the imputation methods yielded significant results, but PM and AV performed better than the others.

**Table 4:** Adjusted Hazard Ratios With 95% CI for CVD Risk: Time-Dependent Cox Model.

Model	Complete Data	LD	Mean	AV	MI	PM (10th percentile)	PM (25th percentile)	PM (50th percentile)
1	**1.04(0.95–1.15)**	1.03(0.92–1.15)	1.02(0.90–1.15)	1.03(0.92–1.15)	0.92(0.73–1.16)	0.83(0.71–0.97)	0.82(0.68–0.98)	0.81(0.68–0.96)
2	**1.04(0.95–1.15)**	1.05(0.92–1.18)	1.02(0.89–1.16)	1.07(0.96–1.20)	1.00(0.88–1.13)	0.92(0.79–1.08)	0.90(0.78–1.04)	0.91(0.77–1.06)
3	**1.04(0.95–1.15)**	1.04(0.92–1.18)	1.04(0.91–1.18)	1.07(0.96–1.20)	1.01(0.90–1.14)	0.93(0.82–1.06)	0.91(0.79–1.04)	0.91(0.79–1.05)
4	**1.04(0.95–1.15)**	6.98(3.57–13.66)*	13.35(7.36–24.21)*	1.45(1.18–1.79)*	3.26(1.86–5.71)*	2.76(1.69–4.50)*	2.86(1.50–5.48)*	2.50(1.52–4.12)*

**Abbreviations**: LD = listwise deletion; Mean = imputation using the mean; AV = imputation using adjacent value; MI = multiple imputation; PM = pattern mixture.

Complete Data: data with no missing values.

Model 1: data with randomly missing values

Model 2: autoregressive missing

Model 3: autoregressive +gender + age

Model 4: NMAR data.

## Conclusions

We developed four models of missing data, generated from a complete data set, and modeled the missing Scr data across the three SHS examinations. Results varied depending on the imputation technique. For the MAR model with 20–40% missing data, all the imputation methods performed similarly. For the NMAR model, AV performed almost as well as PM, possibly because renal dysfunction progresses over time, so using AV may generate results close to those generated with the complete data set. Using different imputation methods to estimate missing Scr values provided varied results, with some methods overestimating Scr and others underestimating it. No one method was superior to the others across all models and exams.

This finding is reasonable because we used two empirical mechanisms to generate patterns of missing data (MAR and NMAR), and because Scr is a protein that changes over time. The PM method performed better in Model 4 across all exams, providing hazard ratio estimates closest to those generated with the complete data set. The PM method outperformed the others on both the mean estimation and the hazard ratio, providing estimates that were closest to those made with the complete data set. Finally, these findings suggest that the PM method for imputing missing Scr values performed better for the data not MAR. Most imputation methods work well when data are MAR. For data not MAR, the PM method performed best for imputing renal function data in this large study of progressive CKD and CVD.

These findings reinforce the point that remedial methods chosen to manage missing values are dependent on the specific case, the nature of the missing data, the nature of the random variable, and the correlation between the missing data and the values in the data. In this case, because of the reasonable assumption that the missing data primarily arose from deterioration in kidney function and resulting mortality (i.e., not MAR), the PM method addressed the missing value problem the best. The assumption of the cause of mortality is as important as the other factors in choosing a remedial method. If mortality is a potential cause of missing values and is not correlated with the variable to be imputed, then the missing values are more appropriately treated as MAR. In that case, methods such as MI or auto-regression are more appropriate.

Further issues may arise regarding the specification of the model used for the imputation. The researcher must exercise judgment regarding whether all factors and covariates that affect the variable to be imputed are controlled for in the regression model. If all the factors are not available, then the researcher must decide whether to impute them based on partial information or use listwise deletion.

This study is strengthened by its large cohort, which allowed us to model missing data, use several covariates to explain the missing data, and generate several types of missing data. This work provides a basis for handling missing data by identifying whether the data are MAR or NMAR.

When the missing data mechanism is not accounted for when performing statistical analyses, the resulting estimates can be misleading. The type and extent of missing data should be considered when choosing an imputation technique. Taking steps to determine whether data are MAR or are missing because of some mechanism can help investigators select the best imputation method for their data.
